# Protective effect of exercise on metabolic dysfunction-associated fatty liver disease: Potential epigenetic mechanisms (Review)

**DOI:** 10.3892/ijmm.2025.5587

**Published:** 2025-07-15

**Authors:** Yanhua Zhang, Yuqin Wei, Huan Liu, Yanju Guo

**Affiliations:** 1 School of Journalism and Communication, Wuhan Sports University, Wuhan, Hubei 430000, P.R. China; 2 School of Physical Education and Health, Hubei Business College, Wuhan, Hubei 430000, P.R. China; 3 College of Sports Medicine, Wuhan Sports University, Wuhan, Hubei 430000, P.R. China; 4 College of Physical Education and Health, Guangxi Normal University, Guilin, Guangxi 541000, P.R. China

**Keywords:** MAFLD, epigenetics, exercise, physical activity

## Abstract

Metabolic dysfunction-associated fatty liver disease (MAFLD) is the most prevalent cause of chronic liver disease worldwide and poses a major health burden that is closely linked to obesity, insulin resistance and type 2 diabetes. While extensive research has elucidated key molecular drivers, no pharmacological therapies have been approved. Emerging evidence highlights the transformative role of physical exercise as a potent nonpharmacological intervention capable of inducing durable metabolic improvements. Epigenetic remodeling, which encompasses DNA methylation, histone modifications and non-coding RNA regulation, has been increasingly recognized as a key mechanism driving these long-lasting effects. Aberrant epigenetic modifications disrupt hepatic lipid metabolism, mitochondrial function, autophagy, inflammation and fibrosis progression, thereby driving MAFLD pathogenesis. The present review comprehensively covers the current knowledge on epigenetic mechanisms implicated in MAFLD and systematically assesses how distinct exercise modalities reshape these epigenetic landscapes to restore hepatic metabolic homeostasis. Understanding the epigenetic underpinnings of exercise-induced hepatic protection offers a promising avenue for advancing personalized interventions and novel therapeutics for MAFLD.

## 1. Introduction

Metabolic dysfunction-associated fatty liver disease (MAFLD) is the leading cause of chronic liver disease worldwide and affects nearly 25% of the population, with disproportionately higher prevalence in developed nations (1). The pathogenesis of MAFLD is multifactorial and driven by a complex interplay among genetic predisposition, metabolic derangements and environmental influences (2-4). Central to its development is insulin resistance, which promotes lipolysis in the adipose tissue and increases free fatty acid flux to the liver (5). Without effective treatment, MAFLD may progress to metabolic dysfunction-associated steatohepatitis (MASH), which requires secondary insults characterized by mitochondrial dysfunction, oxidative stress and lipotoxic injury (6). Furthermore, MAFLD, a hepatic manifestation of the metabolic syndrome, is closely associated with obesity, insulin resistance and type 2 diabetes (7). Critically, MAFLD independently elevates the risk and exacerbates the severity of cardiovascular diseases, particularly coronary artery disease, thereby posing a significant burden on public health systems (8).

Despite decades of research elucidating these multifaceted mechanisms, no pharmacological therapy has been approved for MAFLD treatment (9). Nonetheless, physical exercise has emerged as a remarkably potent intervention, demonstrating efficacy across various stages of MAFLD (10). Unlike pharmacological approaches that typically target single pathways, exercise exerts pleiotropic benefits across the spectrum of MAFLD pathogenesis through an orchestra of adaptive responses that remodel cellular metabolism and signaling networks (11). Importantly, the benefits of exercise persist beyond the intervention period, suggesting induction of long-lasting metabolic reprogramming (12). This sustained effect may be mediated by durable epigenetic modifications that alter the transcriptional landscape of key metabolic and inflammatory genes. Accumulating evidence has implicated epigenetic remodeling as a key mechanism underlying the lasting impact of exercise on hepatic function (13-16).

Epigenetic modifications are heritable and reversible changes in gene expression that occur without altering the underlying DNA sequences. These modifications regulate gene activity through several interrelated mechanisms, including DNA methylation, post-translational modifications of histone proteins (such as methylation and acetylation), chromatin remodeling and regulatory functions of non-coding RNAs (ncRNAs) (17). Collectively, these epigenetic processes influence chromatin architecture and transcriptional activity by modulating the accessibility of genomic regions to transcription factors and their associated cofactors, thereby governing the efficiency and specificity of gene transcription initiation and elongation (18-20). Substantial evidence has demonstrated that epigenetic modifications play a decisive role in MAFLD pathogenesis by dysregulating key pathological processes including hepatic metabolic homeostasis, inflammatory responses and fibrotic progression (21-23). Physical activity can dynamically modulate the epigenetic landscape, restore metabolic flexibility and potentially reverse disease-associated alterations (24,25). Understanding these molecular mechanisms may enable personalized exercise recommendations. The present review comprehensively summarizes current research on how epigenetic dysregulation contributes to MAFLD progression. Based on clinical and preclinical evidence, it was systematically assessed how distinct exercise modalities improve hepatic pathology through epigenetic mechanisms.

## 2. Epigenetics: An intricate yet precise regulatory network

Epigenetics, originally conceptualized by Waddington in 1942, refers to the heritable and reversible changes in gene expression that occur without altering the underlying DNA sequence (26). Epigenetic regulation of gene expression is mediated by three primary mechanisms: DNA methylation, histone modifications and ncRNAs. These mechanisms collectively establish a dynamic regulatory framework that enables the precise control of transcriptional programs (27).

### DNA methylation

DNA methylation is the most extensively studied epigenetic modification in eukaryotic cells. It entails the covalent attachment of a methyl group to the fifth carbon of cytosine residues, primarily within cytosinephosphate-guanine (CpG) dinucleotides, resulting in the formation of 5-methylcytosine (5mC) (28,29). CpG sites, which are characterized by cytosine followed by guanine in the DNA sequence, are particularly prone to methylation (30). This stable and heritable epigenetic marker is strongly associated with transcriptional repression and plays a pivotal role in gene silencing. In addition to its well-established role in gene silencing, DNA methylation is critically involved in chromatin organization, maintenance of genomic stability, and key developmental processes such as X-chromosome inactivation, genomic imprinting and gametogenesis (31-33). Dynamic regulation of DNA methylation is orchestrated by two enzyme families: DNA methyltransferases (DNMTs) and ten-eleven translocation (TET) dioxygenases (34,35). Among the five known DNMTs, DNMT1, DNMT3A and DNMT3B function as the principal catalytic enzymes (36). DNMT1 maintains methylation patterns during DNA replication by recognizing hemi-methylated CpG sites, whereas DNMT3A and DNMT3B mediate *de novo* methylation during early development and lineage specification (37). By contrast, active DNA demethylation is orchestrated by TET enzymes, a Fe (II)- and α-ketoglutarate-dependent dioxygenases, that catalyze the iterative oxidation of 5mC to 5-hydroxymethylcytosine, 5-formylcytosine and 5-carboxylcytosine. These oxidized intermediates facilitate base excision repair via thymine DNA glycosylases, thereby enabling rapid DNA demethylation. Mammals express three TET paralogs (TET1-3) with distinct tissue distributions: TET2 and TET3 dominate in hematopoietic lineages, whereas TET1 initiates oxidative demethylation in developmental contexts (38).

Beyond the nuclear genome, accumulating evidence has revealed that mitochondrial DNA (mtDNA) is also subject to methylation modifications (39). Unlike nuclear DNA, mtDNA is circular, histone-free and organized into compact nucleoid structures (40). High-resolution mapping techniques confirmed the presence of methylation marks within mtDNA, particularly in the D-loop control region, which is a critical site governing replication and transcription initiation (41). Intriguingly, mtDNA methylation occurs not only at CpG sites but also at non-CpG sites, exhibiting a strand-specific bias predominantly affecting the light strand. This pattern diverges from the canonical nuclear methylation landscapes, suggesting specialized regulatory functions within the mitochondria (42).

### Histone modifications

Post-translational modifications of histones represent a major mechanism for dynamically controlling chromatin structure and function (43,44). Highly conserved histone proteins undergo several types of covalent modifications, including acetylation, phosphorylation, methylation, ubiquitination, SUMOylation and ribosylation (45). Among these, the critical roles of histone acetylation and methylation have been particularly well characterized in modulating higher-order chromatin organization and transcriptional regulation (43,46).

Histone methylation primarily occurs at lysine residues in histones. The most studied histone methylation sites are located in histone 3, lysine corresponding to positions 4, 9, 27, 36 and 79 (H3K4, H3K9, H3K27, H3K36, and H3K79 respectively) and histone 4 K20 (H4K20) (47). Lysine residues can undergo mono-, di-, or trimethylation (48). The biological impact of these modifications varies based on the chromatin localization and the specific cell type or developmental stage (49). In general, methylation of histone residues H3K4, H3K36 and H3K79 is linked to transcriptional activation, whereas methylation of H3K9, H3K27 and H4K20 is typically associated with transcriptional repression and the formation of inactive chromatin states (48). However, enrichment of H3K9 trimethylation has been observed in the transcriptionally active euchromatic regions of genes, demonstrating that the impact of histone methylation on gene transcription is complex and nuanced (50). The epigenetic landscape of histone methylation is tightly regulated by the coordinated activities of histone methyltransferases (HMTs) and demethylases (HDMs). HMTs catalyze the transfer of methyl groups from S-adenosylmethionine to specific lysine residues on histone tails, thereby modulating chromatin structure and gene expression (51,52). The most extensively studied HMTs include the SET domain-containing lysine methyltransferase (KMT) family and SET domain-lacking Dot1 family. KMTs typically possess a conserved SET domain that is broadly preserved across species, despite differences in substrate specificity and enzymatic function. Despite their conservation, HMTs exhibit distinct substrate specificities and catalytic functions (53). Histone methylation was traditionally considered a stable modification until the discovery of lysine-specific demethylase 1 (LSD1), which revealed the existence of histone demethylation (54,55). HDMs are broadly categorized into two mechanistically distinct families. The amine oxidase family (for example, LSD1/KDM1A) removes mono- and di-methyl marks through flavin adenine dinucleotide (FAD)-dependent oxidative deamination, while JmjC domain-containing hydroxylases employ α-ketoglutarate and Fe^2+^ to catalyze the removal of diverse modifications including trimethylation (56).

Histone acetylation is a widely studied epigenetic modification, that plays a crucial role in regulating stem cell pluripotency, integrating metabolic signals, and forming a cross-regulatory network with DNA and histone methylation (57-59). The dysregulation of histone acetylation and deacetylation is commonly associated with various diseases, including cancer, neurodegenerative diseases and cardiovascular diseases (60-62). The most common sites of histone acetylation include lysine residues at positions 9, 14, 18 and 23 of histone H3 (H3K9, H3K14, H3K18 and H3K23, respectively), and at positions 8, 12, 16, and 20 of histone H4 (H4K8, H4K12, H4K16 and H4K20) (63). Generally, histone acetyltransferases (HATs)-mediated acetylation weakens the interaction between histones and DNA by neutralizing positive charges on histone tails, thereby promoting chromatin opening and transcriptional activation (64). Based on their catalytic mechanisms and cellular localization, HATs can be classified into two types: HAT A and HAT B. Members of the HAT A family are located in the nucleus, where they transfer acetyl groups to lysine residues after histone assembly into nucleosomes. By contrast, members of the HAT B family function in the cytoplasm by transferring acetyl groups to free histones before becoming nucleosomes (65). Histone deacetylases (HDACs) are transcriptional repressors that remove acetyl groups and restore tight binding between histones and DNA. HDACs are categorized into four classes (I-IV) based on their structural characteristics and coenzyme requirements. Classical HDACs (Class I/II/IV) are activated by Zn^2+^, whereas sirtuins (SIRTs)-related HDACs (Class III) are activated by NAD^+^ (66). Current research is focused on understanding the complex mechanisms of these enzymes, their roles in chromatin remodeling, and their potential as therapeutic targets in various diseases.

### NcRNAs

In addition to DNA and histone modifications, ncRNAs have emerged as key epigenetic regulators of gene expression and chromatin organization. The human genome is pervasively transcribed, with ~75% DNA generating RNA molecules and only 2% encoding proteins. The remaining transcriptional output comprises ncRNAs that function directly as RNA entities to orchestrate complex biological processes (67). Emerging advances in epigenomics and high-throughput sequencing have identified more than 200,000 ncRNA species, establishing ncRNAs as indispensable regulators of cellular homeostasis and disease pathogenesis (68). ncRNAs are broadly classified into two major categories based on their structural or functional roles: Structural and regulatory ncRNAs. Structural ncRNAs, such as ribosomal RNA, transfer RNA, small nuclear RNAs and small nucleolar RNAs, are integral to fundamental cellular processes including ribosome assembly, protein translation and mRNA splicing. Regulatory ncRNAs, including microRNAs (miRNAs or miRs), long non-coding RNAs (lncRNAs), small interfering RNAs, PIWI-interacting RNAs and circular RNAs, primarily modulate gene expression via epigenetic, transcriptional, or post-transcriptional mechanisms (69). Certain ncRNAs such as miRNAs, small nucleolar RNAs and circular RNAs (circRNAs), demonstrate evolutionary conservation across species, whereas others, particularly lncRNAs, frequently lack sequence homology. Accumulating evidence has indicated that the expression profiles of circRNA-producing genes, circular-to-linear transcript ratios, and splice isoform patterns of circRNAs are highly cell type-specific (70-73). These observations underscore that circRNA biogenesis is not a random byproduct, but rather a conserved and tightly regulated facet of gene expression.

Among regulatory ncRNAs, miRNAs are ~22-nucleotidelong molecules that primarily function through post-transcriptional gene silencing. By binding to complementary sequences within the 3′ untranslated regions of target mRNAs, miRNAs mediate mRNA degradation or translational repression, thereby modulating diverse cellular processes such as proliferation, differentiation and apoptosis (74,75). Notably, miRNAs can be selectively incorporated into extracellular vesicles (EVs), including exosomes, and secreted into the extracellular environment to facilitate intercellular communication (76). These vesicle-containing miRNAs facilitate intercellular communication by transferring regulatory information to recipient cells, thereby influencing tissue homeostasis and contributing to disease pathogenesis, particularly in cancer, metabolic disorders and inflammatory diseases (77-79). lncRNAs, defined as transcripts longer than 200 nucleotides, display versatile mechanisms of action at the epigenetic, transcriptional and post-transcriptional levels. They serve as molecular scaffolds by recruiting chromatin-modifying complexes to specific genomic loci, thereby altering chromatin accessibility and influencing gene expression (80). lncRNAs may also act as molecular decoys, sequestering transcription factors or miRNAs to modulate downstream signaling pathways. Furthermore, some lncRNAs function as guides that direct regulatory proteins to their genomic targets or as enhancers that promote transcription by stabilizing chromosomal looping (81). Through these multifaceted mechanisms, lncRNAs critically regulate cellular identity, stress responses and disease development, highlighting their significance as potential therapeutic targets and biomarkers in various pathological conditions (82).

## 3. Molecular mechanism underlying epigenetic regulation in MAFLD

**Figure 1:**
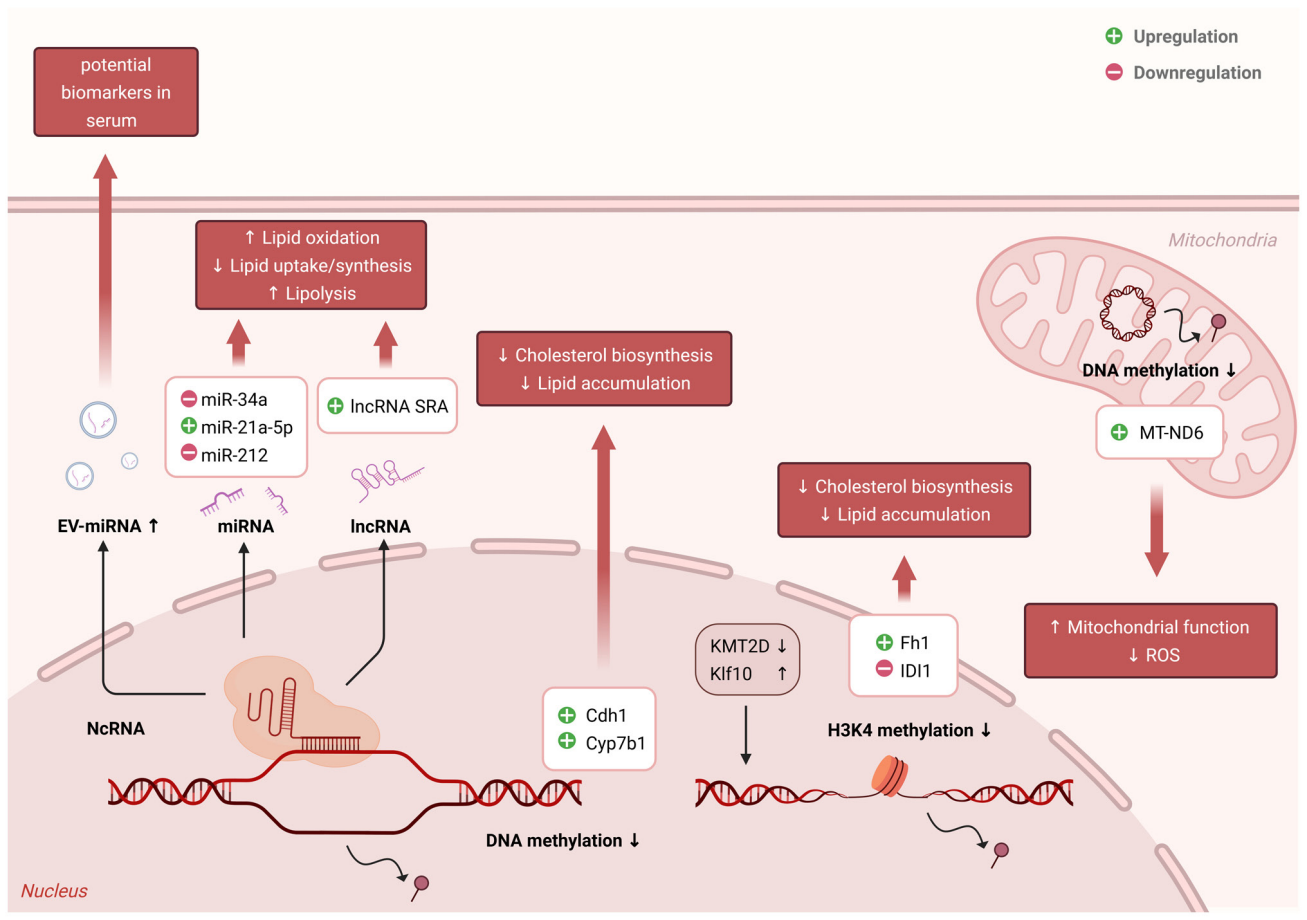
Epigenetic mechanisms contribute to the pathogenesis of metabolic dysfunction-associated fatty liver disease. lncRNA, long non-coding RNA; miRNA or miR, microRNA; ROS, reactive oxygen species; SRA, steroid receptor RNA activator; EV, extracellular vesicle.

Given their dynamic and reversible nature, epigenetic modifications act as key mediators that link environmental factors to long-term changes in gene expression (83). As a central metabolic organ, the liver is particularly responsive to such epigenetic influences (17). Increasing evidence indicates that the dysregulation of epigenetic mechanisms (namely DNA methylation, histone modifications and ncRNAs) plays a pivotal role in the development and progression of MAFLD (23,84,85). These alterations affect the genes involved in lipid metabolism, insulin signaling, inflammation and fibrogenesis, thereby contributing to hepatic metabolic derangement (86,87). Current research progress was reviewed, focusing on three major epigenetic mechanisms and their specific contributions to MAFLD development (Fig. 1).

### DNA methylation in MAFLD pathogenesis

In MAFLD, both global and locus-specific hypermethylation has been observed in genes that regulate lipid metabolism, mitochondrial function and inflammation (88,89). For example, hypermethylation of the peroxisome proliferator-activated receptor gamma coactivator 1-alpha (PGC-1α) promoter leads to decreased expression of this master regulator of mitochondrial biogenesis, contributing to impaired fatty acid oxidation and increased lipid accumulation (90,91). Similarly, the hypermethylation of mitochondrially encoded NADH dehydrogenase 6 (MT-ND6) correlates with decreased gene expression and disrupted oxidative phosphorylation, underscoring the impact of methylation on mitochondrial dysfunction in MAFLD (92). Genes involved in fibrogenesis, such as transforming growth factor beta 1 and platelet-derived growth factor alpha, show hypomethylation in MASH, facilitating their overexpression and promoting fibrotic remodeling (93). By contrast, tumor suppressor genes such as suppressor of cytokine signaling 1 and Ras association domain family member 1 often exhibit promoter hypermethylation, silence protective mechanisms and enabling disease progression (94). Epigenetic reprogramming also affects genes associated with insulin sensitivity and lipid homeostasis, including dipeptidyl peptidase 4, ATP citrate lyase and acyl-CoA synthetase long-chain family member 4 (95-98). These modifications not only contribute to hepatic lipid dysregulation but also influence systemic metabolic pathways.

### Histone modifications and chromatin remodeling in MAFLD pathogenesis

In MAFLD, aberrant histone acetylation and methylation patterns have been linked to the altered transcription of genes involved in lipid metabolism and inflammation (99). HDAC3 is a crucial regulator of hepatic lipid homeostasis. Loss of HDAC3 activity has been associated with an increased expression of lipogenic transcription factors, exacerbating steatosis (100,101). The SIRT family, comprising NAD^+^-dependent deacetylases such as SIRT1, SIRT3 and SIRT6, plays a protective role against MAFLD. SIRT1 deacetylates and inhibits sterol regulatory element-binding protein-1c (SREBP-1c) and carbohydrate-responsive element-binding protein (ChREBP), thereby suppressing *de novo* lipogenesis (102). It also enhances fatty acid oxidation and reduces inflammation by deacetylating histones at the promoters of metabolic and inflammatory genes, such as nuclear factor-kappa B (NF-κB) and peroxisome proliferator-activated receptor alpha (PPARα) (103). SIRT3 maintains mitochondrial integrity and antioxidant defense, whereas SIRT6 modulates gluconeogenesis and lipid synthesis (104). Perturbations in these SIRTs compromise hepatic metabolic resilience (105). Histone methylation also contributes to MAFLD pathology. Elevated levels of repressive markers such as H3K9me2 and H3K36me3 are observed in MASH, correlating with the silencing of genes involved in hepatic steatosis (106). Conversely, decreased levels of activating markers such as H3K4me3 may impair the expression of genes necessary for lipid catabolism. Enzymes such as PR domain-containing protein, which regulates these histone marks, are dysregulated in susceptible populations, indicating a potential role in MAFLD susceptibility and progression (107).

### NcRNAs as epigenetic regulators in MAFLD pathogenesis

NcRNAs, including miRNAs and lncRNAs, are increasingly being recognized as key epigenetic regulators of MAFLD. These molecules modulate gene expression post-transcriptionally and often function as critical nodes in metabolic and inflammatory networks (108). Among miRNAs, microRNA-122 (miR-122) is a highly conserved miRNA that is predominantly expressed in the liver and plays a critical role in the regulation of liver metabolism (109). Its downregulation in the liver tissue leads to disrupted lipid metabolism, increased inflammation and fibrosis (110,111). Paradoxically, circulating miR-122 levels were elevated in the early stages of MAFLD and MASH, likely due to enhanced secretion and hepatocellular injury-induced leakage from hepatocytes, despite the significant downregulation of hepatic miR-122 expression observed in these patients (112,113). miR-34a is expressed at low levels in hepatocytes and exerts potent regulatory control over hepatic lipid metabolism (114). It is markedly upregulated in both the liver and circulation of patients with MASH and serves as a reliable biomarker of disease severity (115). Functionally, miR-34a directly targets key metabolic regulators including SIRT1 and PPARα, thereby impairing fatty acid oxidation and promoting lipogenesis and steatotic progression (116). Moreover, miR-34a suppresses hepatocyte nuclear factor 4 alpha and attenuates AMP-activated protein kinase alpha (AMPKα) signaling, further exacerbating lipid accumulation and hepatocellular dysfunction (116,117). miR-21, a stress-responsive miRNA, is markedly elevated in the liver and circulation of patients with MASH and plays multifaceted roles in hepatic pathophysiology (118). In addition to its well-established oncogenic potential, miR-21 contributes to steatosis, inflammation and fibrosis by targeting key metabolic and regulatory pathways (118). It suppresses phosphatase and tensin homolog (PTEN), thereby enhancing *de novo* lipogenesis and fatty acid uptake, and downregulates PPARα, limiting lipid oxidation (119,120). Additionally, miR-21 modulates the HMG-box transcription factor 1-p53-sterol regulatory element-binding protein 1 axis, promotes hepatic stellate cell (HSC) activation, and collectively exacerbates liver inflammation and fibrogenesis (121). Additional miRNAs such as miR-192, miR-193a and miR-33 further modulate pathways involving TGF-β, AMPK and cholesterol efflux (122). Moreover, circulating miRNAs are emerging as promising non-invasive biomarkers for MAFLD diagnosis and risk stratification. Notably, miR-34a-5p has been incorporated into the NIS4 diagnostic algorithm, a blood-based composite panel, to detect MASH (123). Additionally, circulating miRNAs, such as miR-99a and miR-192, are also elevated in MAFLD and are associated with disease severity, highlighting their potential utility in monitoring disease progression (123-125). lncRNAs critically regulate glucose and lipid metabolism in MAFLD via diverse nuclear and cytoplasmic mechanisms. lncRNAs such as HOX antisense intergenic RNA and maternally expressed gene 3, promote gluconeogenesis and insulin resistance by targeting SIRT1, forkhead box O1, and microRNA-214/139 (126-128). Others, including brown-fat long ncRNA 1, metastasis-associated lung adenocarcinoma transcript 1, lnc RNA Gm15622, and lncRNA H19, enhance hepatic steatosis via the activation or stabilization of SREBP-1c and its downstream targets (129-132). Conversely, protective lncRNAs such as lncRNA suppressor of hepatic gluconeogenesis and lipogenesis, lncRNA hepatic regulator 1, and macrophage inflammation-suppressing RNA transcript 2 improve metabolic profiles by suppressing lipogenesis or restoring deacetylase activity (133-135). These findings highlight lncRNAs as central modulators of MAFLD progression and promising therapeutic targets.

## 4. Exercise and its impact on MAFLD

Epigenetic research has provided critical insights into the molecular underpinnings of MAFLD, revealing the roles of DNA methylation, histone modifications and ncRNAs in the regulation of hepatic lipid metabolism, inflammation and fibrogenesis. However, MAFLD is a multifactorial disease, and its effective prevention and treatment require a broader understanding. Lifestyle interventions, particularly physical activity, have gained increasing attention as essential components in the management of MAFLD. Physical activity is a key modulator of hepatic and systemic metabolic health (136). Structured lifestyle interventions, such as increasing daily step counts and leveraging smart-device-guided exercise programs, have been shown to enhance cardiopulmonary fitness, improve metabolic health, and promote overall physiological well-being in high-risk populations (137,138). In addition, exercise modulates gene expression profiles and elicits widespread adaptive responses, including increased endurance, enhanced efficiency of the respiratory and cardiovascular systems, and regulation of immune and neurophysiological processes (139-141). Physical inactivity is a well-established risk factor for the onset of chronic diseases, largely because of its association with adverse molecular and biochemical alterations in specific tissues (142). To elucidate the therapeutic effect of physical activity on MAFLD, the following sections review the distinct physiological and molecular effects of various exercise modalities and highlight their shared and unique mechanisms for mitigating hepatic steatosis and metabolic dysfunction.

### Aerobic exercise and MAFLD improvement

Aerobic exercise, defined as sustained rhythmic physical activity that primarily depends on oxidative metabolism, is a fundamental component of a healthy lifestyle. Regular aerobic exercise elicits a wide array of systemic benefits, including enhanced cardiovascular function, improved glucose metabolism, optimized lipid profiles, and reduced systemic inflammation (143-146). In this context, aerobic exercise has gained prominence as a potent non-pharmacological intervention to prevent and ameliorate MAFLD (147). Clinically, aerobic exercise has been shown to significantly reduces liver enzyme levels (ALT and AST), decreases intrahepatic fat accumulation, and improves insulin resistance, even in the absence of significant weight loss (148). Mechanistically, aerobic exercise enhances hepatic fatty acid β-oxidation while suppressing *de novo* lipogenesis by downregulating lipogenic transcription factors such as SREBP-1c and upregulating PPARα (147,149). This shift in lipid metabolism reduces intrahepatic fat accumulation independent of weight loss (150). Concurrently, aerobic exercise attenuates hepatic oxidative stress by enhancing the expression of antioxidant enzymes such as superoxide dismutase, catalase and glutathione peroxidase, thereby mitigating reactive oxygen species-mediated damage (151). Inflammation, a critical driver of MAFLD progression, is also alleviated by aerobic exercise through the downregulation of pro-inflammatory cytokines such as tumor necrosis factor-alpha (TNF-α) and interleukin-1 beta (152-154). Additionally, aerobic exercise enhances hepatic autophagy, particularly lipophagy-a selective autophagic process essential for lipid droplet clearance and energy mobilization (24). Through activation of the AMPK-PPARα signaling axis, aerobic training promotes mitochondrial fatty acid oxidation, mitigates endoplasmic reticulum (ER) stress, and reduces fibrotic markers including α-smooth muscle actin and TNF-α (149,155,156). Notably, aerobic exercise significantly improves mitochondrial quality, a key pathological deficit in MAFLD, by promoting mitochondrial biogenesis, augmenting mitophagy via PTEN-induced kinase 1/Parkin RBR E3 ubiquitin protein ligase signaling, and maintaining oxidative phosphorylation (150,157). Collectively, these adaptations underscore the multifaceted therapeutic potential of aerobic exercise for halting or reversing MAFLD progression.

### Resistance training and its role in MAFLD management

Resistance exercise, also known as strength training, involves repeated muscle contractions against external loads to enhance muscle strength, endurance and mass. Unlike aerobic exercise, which primarily targets cardiovascular fitness and oxidative metabolism, resistance training predominantly affects the neuromuscular system by inducing metabolic adaptations through mechanical loading, muscle hypertrophy and functional remodeling (158,159). An increasing body of evidence highlights that resistance exercise confers substantial systemic benefits, including improved insulin sensitivity, enhanced glucose uptake, favorable modulation of lipid profiles, increased resting energy expenditure, and attenuation of chronic low-grade inflammation (146,160,161). From a metabolic perspective, resistance exercise stimulates skeletal muscle glucose transporter type 4 translocation, promotes glycogen synthesis, and enhances mitochondrial oxidative capacity, thereby improving peripheral glucose disposal and energy metabolism (162). In addition, resistance training stimulates the secretion of muscle-derived cytokines (myokines), such as irisin and interleukin-6, which exert endocrine effects by regulating lipid metabolism, suppress inflammatory responses, and promoting systemic metabolic homeostasis (163). Resistance exercises have emerged as an effective and accessible intervention for MAFLD. Clinical studies have consistently demonstrated that regular resistance training significantly reduces the intrahepatic triglyceride content, improves insulin sensitivity, lowers serum liver enzyme levels (ALT and AST), and reduces visceral adiposity (164,165). Notably, simple bodyweight-based exercises such as squats and push-ups have been shown to ameliorate hepatic steatosis and improve metabolic parameters without the need for specialized equipment, thus enhancing their feasibility and applicability across diverse patient populations (166). At the molecular level, resistance exercise improves hepatic lipid metabolism by activating the AMPK signaling pathway, upregulating PPARα, and downregulating lipogenic transcription factors such as SREBPs (155). Although resistance training and aerobic exercise exhibit distinct physiological targets, emerging evidence suggests that combining both modalities yields synergistic benefits, achieving greater reductions in intrahepatic fat, systemic insulin resistance, and body fat percentage than either intervention alone. Nevertheless, even when applied independently, resistance exercise confers significant hepatoprotective effects and represents a vital component of lifestyle interventions aimed at mitigating MAFLD progression (167).

### Comparative effects of high-intensity interval training (HIIT) and moderate-intensity continuous training (MICT)

HIIT, typically performed at 70-90% of the maximal heart rate, is characterized by brief bouts of vigorous effort that substantially elevate cardiovascular demand and metabolic rate (168). HIIT, a widely adopted form of high-intensity exercise, has been shown to rapidly improve cardiorespiratory fitness, enhance insulin sensitivity, promotes mitochondrial biogenesis, and elicits potent anti-inflammatory responses (169-171). These adaptations contribute to significant reductions in the risk of metabolic disorders such as type 2 diabetes, cardiovascular disease and MAFLD (172). MICT, typically conducted at 45-65% of the maximal heart rate, emphasizes sustained aerobic activity over longer durations. Although the metabolic stimulus is less intense than in HIIT, MICT effectively enhances lipid metabolism, improves glycemic control, reduces visceral adiposity, and supports cardiovascular health (168,172). It is particularly valued for its high safety profile, broad accessibility, and strong patient adherence, making it a preferred strategy for populations with limited exercise capacity or comorbidities (173). Longitudinal cohort studies have revealed that sustained engagement in vigorous physical activity from young adulthood significantly lowers the risk of developing MAFLD in midlife (174). Similarly, clinical and experimental research have shown that HIIT more effectively reduces intrahepatic triglyceride accumulation, improves glucose metabolism, and attenuates hepatic inflammation and fibrosis than MICT (175,176). Mechanistically, HIIT enhanced hepatic fatty acid oxidation, suppressed lipogenesis, and reduced the infiltration of pro-inflammatory monocyte-derived macrophages into the liver. Moreover, high-intensity exercise has been associated with favorable modulation of systemic inflammatory markers, such as C-reactive protein, whereas light-intensity physical activity appears insufficient to elicit such benefits (177). Notably, HIIT robustly activates hepatic fatty acid oxidation pathways and upregulates mitochondrial oxidative enzymes such as carnitine palmitoyl-transferase 1 alpha and β-hydroxy-acyl-CoA dehydrogenase, while promoting mitochondrial biogenesis via PGC-1α induction. These adaptations suppress hepatic lipogenesis and mitigate ER stress by downregulating the protein kinase R-like ER kinase-activating transcription factor 4-C/EBP homologous protein signaling pathway, a critical driver of hepatocellular dysfunction under lipotoxic conditions (178). MICT moderately enhances antioxidant capacity and downregulates hepatic CHOP expression, contributing to the partial relief of ER stress and hepatic lipid deposition (178). Although both MICT and HIIT reduce hepatic steatosis, HIIT often achieves more rapid and profound metabolic improvements, including improved insulin sensitivity and mitochondrial function (174). Nonetheless, moderate-intensity exercise remains a valuable strategy, particularly for individuals with lower baseline fitness or comorbidities, offering consistent and slightly lesser improvements in hepatic fat content and metabolic parameters. In conclusion, both exercise intensities confer substantial health benefits. However, the choice of intensity should be individualized based on patient characteristics, goals and tolerance.

## 5. Epigenetic mechanisms underlying exercise-mediated protection in MAFLD

Given the established roles of both epigenetic regulation and physical activity in MAFLD, increasing attention has been directed toward understanding how these two factors may interact. Although this field remains in its early stages, increasing evidence supports a mechanistic link between physical activity and epigenetic remodeling in MAFLD (179). To investigate the epigenetic mechanisms underlying exercise-mediated amelioration of MAFLD, PubMed (https://pubmed.ncbi.nlm.nih.gov/) was systematically searched using the following query: ['Non-alcoholic Fatty Liver Disease' (Mesh) OR MAFLD OR NASH OR MAFLD OR 'non-alcoholic fatty liver' OR steatosis] AND ['Exercise'(Mesh) OR 'Exercise Therapy' (Mesh) OR 'Physical Exertion' (Mesh) OR 'physical activity' OR 'aerobic exercise' OR 'resistance training'] AND ['Epigenesis, Genetic' (Mesh) OR 'DNA Methylation' (Mesh) OR 'Histone Code' (Mesh) OR 'RNA, Untranslated' (Mesh) OR epigenetic^*^ OR 'DNA methylation' OR 'histone modification^*^' OR 'non-coding RNA' OR miRNA OR lncRNA]. All the retrieved records were combined, deduplicated and rigorously screened to identify studies in which exercise served as the primary intervention. Studies were further prioritized based on the explicit mechanistic exploration of exercise-induced epigenetic modifications (Table I).

### Exercise-induced DNA methylation in MAFLD

Exercise is a potent regulator of DNA methylation dynamics, influencing gene expression and cellular function (180). Notably, global DNA methylation tends to increase with age, leading to the dysregulation of gene expression and physiological deterioration (181). However, compared with sedentary individuals, those engaged in sustained physical activity exhibit a significantly slower progression of DNA methylation alterations (16). Exercise has been shown to reduce the plasma levels of folate and vitamin B12, two essential cofactors for one-carbon metabolism, thereby indirectly influencing DNA methylation stability (182,183). Notably, considerable divergence remains in the literature regarding the overall directionality of methylation changes induced by exercise. For instance, a study assessing trained young male cyclists following an acute aerobic exercise bout (4 min at 75% VO_2_ max followed by a 15-min time trial) reported a significant decrease in global DNA methylation levels (184). By contrast, Robson-Ansley *et al* (185) observed no significant changes in global DNA methylation in trained males following acute running exercise (45 min at 60% VO_2_ max followed by a 5-min time trial), suggesting that these changes might be negligible in certain individuals or that gains and losses in methylation could be balanced at the global level. These discrepancies may be attributed to differences in exercise modalities, sample sources and detection methodologies. Given the ongoing debate surrounding genome-wide methylation analyses, investigations into the epigenetic effects of exercise on metabolically active organs, such as the liver, may offer more physiologically relevant insights. Pirola *et al* (92) highlighted the critical role of mitochondrial DNA methylation in MAFLD. They demonstrated that the mitochondrial-encoded gene MT-ND6 exhibited significant hypermethylation in patients with MAFLD, leading to its transcriptional repression and correlation with disease severity. Importantly, the aforementioned study demonstrated that higher physical activity is associated with reduced hepatic MT-ND6 methylation, suggesting that exercise may enhance mitochondrial function and metabolic homeostasis via mitochondrial DNA methylation (92). Zhou *et al* (186) reported that a high-fat diet induced widespread pathological hypermethylation of enhancer and promoter regions in the liver, disrupting the expression of genes critical for lipid metabolism. Regular exercise reversed these aberrant DNA methylation changes, restored the expression of key metabolic genes, and restored lipid homeostasis (186). Exercise also influences several regulatory factors that play critical roles MAFLD. For instance, interval walking training was shown to epigenetically regulate the NF-κB2 gene through DNA methylation remodeling (187). In addition, high-intensity exercise has been associated with favorable DNA methylation changes in metabolic regulatory genes, contributing to improved insulin sensitivity and attenuated fatty liver disease (25,188,189). Moreover, increased DNA methylation in the promoter region of nuclear receptor co-repressor 1 has been observed in trained male mice, and this epigenetic modification appears heritable. This finding suggests that exercise may modulate the epigenetic regulation of key genes associated with MAFLD (190). However, their precise role in MAFLD pathogenesis and progression requires further investigation.

### Exercise-induced histone modification in MAFLD

Despite significant advances over the past decade in defining the transcriptional responses of genes to different modes of exercise, the histone modification mechanisms underlying these changes, particularly in hepatic tissues, remain incompletely characterized. Exercise has been shown to trigger histone methylation, acetylation and phosphorylation, which are key regulators of transcription (191,192). However, these studies have largely focused on the role of histone modifications in skeletal muscle fiber type switching and improvement of cognitive function. Exercise promotes histone H3 acetylation at specific promoter regions of metabolic genes, enhance histone H3 phosphorylation to facilitate chromatin relaxation, and induces histone methylation changes associated with transcriptional activation or repression (189). Notably, emerging research has revealed that exercise-mediated regulation of histone methylation may contribute to metabolic disease prevention (193). For example, studies have demonstrated that HIIT alleviates lipid accumulation and inflammation in MAFLD models by reducing H3K4me1 mediated by KMT at the isopentenyl-diphosphate delta isomerase 1 promoter, thereby suppressing cholesterol biosynthesis and improving hepatic metabolic homeostasis (194). Exercise also induces hepatic Krüppel-like factor 10 expression via the cyclic adenosine monophosphate-protein kinase A-cAMP response element-binding protein pathway, which upregulates fumarate hydratase 1 to reduce fumarate accumulation, thereby decreasing H3K4me3 levels at lipogenic gene promoters and ameliorating lipid accumulation, inflammation and fibrosis in MASH (195). Taken together, these findings suggest that exercise modulates histone methylation dynamics at key metabolic gene loci, contributing to the attenuation of hepatic steatosis, inflammation and fibrosis. Exercise has emerged as a potent modulator of the SIRT family members, particularly SIRT1 and SIRT6, which are critical regulators of metabolic homeostasis (196). Regular aerobic, resistance and combined training upregulate hepatic SIRT1 expression in high-fat diet-induced MAFLD models, enhancing insulin sensitivity, reducing hepatic lipid accumulation, and lowering serum liver enzymes (197,198). SIRT1 activation promotes fatty acid oxidation, suppresses *de novo* lipogenesis via deacetylation of SREBP-1c and ChREBP, and inhibits inflammation through NF-κB signaling (199). In parallel, exercise-induced SIRT6 activation enhances mitochondrial biogenesis and shifts muscle fiber composition towards oxidative and fatigue-resistant types, thereby improving systemic metabolic resilience (200). Although the metabolic benefits of SIRT activation are well recognized, the effect of exercise-induced SIRT1 and SIRT6 modulation on hepatic histone modifications in MAFLD remains largely unexplored. SIRTs function as NAD^+^-dependent deacetylases targeting both histone and non-histone proteins to regulate chromatin structure and gene transcription. SIRT1 deacetylates histones H3K9 and H4K16, while SIRT6 targets H3K9 and H3K56, thereby influencing pathways linked to lipid metabolism, inflammation and genomic stability (201,202). However, the mechanism through which exercise-driven SIRT activation reprograms the hepatic epigenome to mitigate MAFLD progression remains unclear. Future studies employing high-resolution epigenomic profiling are needed to delineate the histone marks modulated by SIRT1 and SIRT6 in response to exercise and to uncover their roles in hepatic metabolic adaptation and fibrosis attenuation. Notably, lactate is a principal metabolite of glycolysis and serves as a substrate for mitochondrial respiration, thereby linking the glycolytic and oxidative metabolic pathways (203). During physical exercise and other physiological stresses, elevated lactate levels are released into the circulation, where lactate acts not only as a metabolic intermediate but also as an active signaling molecule (204). Emerging evidence suggests that lactate regulates gene expression via histone lactylation, a novel epigenetic modification that modulates chromatin structure and transcriptional activity. By influencing both transcriptional programs and cellular signaling pathways, lactate contributes to tissue adaptation, metabolic homeostasis, and inflammation control (205). Thus, histone lactylation is a potential mechanism by which exercise promotes cellular and systemic resilience. However, the specific dynamics of histone lactylation in hepatic tissues following exercise, the critical gene targets involved, and their roles in MAFLD resolution remain largely unknown. Future studies should aim to characterize the lactate-histone lactylation axis in the liver to improve understanding of its therapeutic potential in metabolic disease.

### Exercise-induced ncRNAs in MAFLD

With an increasing understanding of the biological functions of ncRNAs, exercise has emerged as a potent non-pharmacological strategy to modulate ncRNA expression and drive systemic adaptation (206). Among ncRNAs, miRNAs serve as highly stable and readily detectable regulators of gene expression across multiple tissues (207). At the tissue level, skeletal muscle responds to exercise with enhanced expression of miRNA-1 and miRNA-133a, facilitating myogenic differentiation and mitochondrial biogenesis through activation of the phosphatidylinositol 3-kinase/protein kinase B and PGC-1α pathways (208,209). Exercise induces the upregulation of miR-21 and miRNA-126 in cardiac tissue, promoting physiological hypertrophy and angiogenesis and attenuating apoptosis (210,211). In adipose tissue, exercise modulates miRNAs such as miR-143, miR-145 and miR-222, favoring lipolysis and improving insulin sensitivity (212-214). Additionally, exercise-induced shifts in miR-132 enhance neurogenesis and cognitive function (215). Collectively, these systemic adaptations underscore the critical role of ncRNA networks in orchestrating the complex, multi-organ benefits of regular physical activity. Among the various organ systems affected by exercise-induced changes in miRNAs, the liver has drawn particular interest, particularly in the context of MAFLD. Exercise ameliorates MAFLD progression via miRNA-mediated mechanisms, notably by restoring the expression of key hepatic miRNAs (216). Specifically, aerobic exercise upregulates miR-33 and miR-122, two miRNAs that are suppressed in MAFLD and whose restoration leads to the inhibition of lipogenic genes such as SREBP1 and PPAR-γ, thereby attenuating hepatic lipid accumulation (217,218). Hypoxic exercise training reduces hepatic miR-378b expression in obese rats, thereby altering the FAS/CPT1A protein ratio and decreasing hepatic fatty acid oxidation, ultimately contributing to reduced fat accumulation and improved lipid profiles in diet-induced obesity (219). Furthermore, exercise downregulates miR-212, whose elevated expression promotes the suppression of fibroblast growth factor-21 (FGF-21) suppression and exacerbates hepatic steatosis (220). Collectively, these findings suggest that exercise-induced modulation of ncRNAs is a promising non-pharmacological strategy for the prevention and treatment of MAFLD, highlighting the therapeutic potential of targeting specific miRNA circuits within the context of metabolic diseases. Accumulating evidence indicates that exercise exerts potent hepatoprotective effects by modulating EV-miRNAs and lncRNAs, thereby coordinating inter-organ metabolic communication and maintaining systemic homeostasis. Aerobic exercise has been shown to remodel the circulating miRNA profile, notably downregulating EV-associated miRNAs such as miR-122, miR-192 and miR-22, leading to enhanced adipose tissue insulin sensitivity and reduced hepatic lipid accumulation (221,222). In parallel, exercise suppresses miR-33, miR-34a and miR-212, alleviating hepatic steatosis by promoting autophagy, inhibiting lipogenesis, and activating metabolic regulators such as FGF-21 (214,220,223). Emerging studies have also highlighted the role of lncRNAs, particularly exercise-induced downregulation of steroid receptor RNA activator, which activates p38 mitogen-activated protein kinase/c-Jun N-terminal kinase signaling and promotes adipose triglyceride lipase-mediated lipolysis, thereby mitigating hepatic inflammation and lipid deposition (224,225). Accumulating data has expanded the regulatory network. Long-term aerobic exercise increases adipose-derived exosomal miR-324, which targets Rho-associated coiled-coil-containing protein kinase 1 to enhance insulin sensitivity and attenuate liver fat accumulation in MAFLD (226). Similarly, exercise upregulates miR-21a-5p, suppressing key lipid metabolism genes such as fatty acid-binding protein 7, 3-Hydroxy-3-methylglutaryl-CoA reductase, acetyl-CoA acetyltransferase 1 and oxidized low-density lipoprotein receptor 1, further linking miRNA-mediated regulation to metabolic improvement (227). Beyond hepatic effects, exercise-induced changes in exosomal miRNAs, such as miR-122-5p, also contribute to vascular remodeling and skeletal muscle angiogenesis (228). However, context-dependent effects have been observed, with elevated miR-122 levels following weight-loss interventions being associated with suboptimal hepatic fat reduction, underscoring the complexity of miRNA-mediated metabolic regulation (218). In addition to altering specific miRNAs, exercise influences the miRNA-processing machinery itself, including the upregulation of Dicer1, an essential RNase involved in miRNA maturation, which may further amplify its impact on miRNA biogenesis and function (227). Moreover, the modulation of thyroid hormone-responsive miRNA networks, such as miR-383, miR-146b and miRNA-200a, provides another mechanism by which exercise restores hepatic lipid metabolism and redox balance (229). Collectively, these findings delineate a sophisticated regulatory network wherein exercise orchestrates small RNA-mediated inter-organ communication, metabolic reprogramming, and the resolution of inflammation. This emerging understanding not only deepens insights into the molecular underpinnings of exercise-induced benefits but also opens new avenues for RNA-based therapeutic strategies targeting metabolic disorders.

### Challenges and perspectives in epigenetic research on exercise and MAFLD

Despite these promising advances, exploration of exercise-induced epigenetic regulation of MAFLD remains in its infancy. Studies have predominantly focused on a limited set of candidate genes or pathways, often relying on bulk tissue analyses without resolving cell-type-specific or spatially restricted epigenetic dynamics. Given the cellular heterogeneity of the liver, future investigations using single-cell epigenomic technologies and spatial transcriptomics are critical for dissecting the differential reprogramming of hepatocytes, Kupffer cells, HSCs and endothelial populations during MAFLD progression and resolution (230). Moreover, although several key epigenetic markers-such as DNA methylation, histone acetylation, and miRNA expression-have been implicated, an integrated, multi-omics understanding of how these layers interact to drive systemic metabolic adaptation is still lacking. Longitudinal studies combining epigenomics, transcriptomics, proteomics and metabolomics are essential to capture the temporal evolution of exercise-induced hepatic reprogramming and identify causal regulatory nodes amenable to therapeutic interventions. Another major limitation is the relative paucity of human studies. Much of the current knowledge has been derived from rodent models, which, while informative, may not fully recapitulate the complexity of human MAFLD pathophysiology, especially when considering genetic, dietary and lifestyle variabilities. Large-scale, well-controlled clinical trials incorporating biopsy-based liver epigenomic profiling before and after structured exercise interventions are urgently needed to validate these mechanistic findings and translate them into actionable clinical insights. Furthermore, most studies have examined the immediate or short-term epigenetic responses to exercise; however, the durability and reversibility of these modifications remain poorly understood (231). Whether exercise-induced epigenetic changes persist after cessation of training, whether they exhibit 'metabolic memory' effects, and to what extent they can counteract pre-existing pathogenic epigenetic signatures are critical questions that warrant rigorous investigation. Emerging evidence has highlighted the potential of exercise to modulate transgenerational metabolic health through epigenetic mechanisms. Maternal exercise during pregnancy protects the offspring against high-fat diet-induced MAFLD by reprogramming hepatic metabolism early in life (190). Mechanistically, this protective effect is associated with the activation of key metabolic pathways, including AMPK, PPARα and PGC1α signaling, resulting in enhanced fatty acid oxidation, suppressed lipogenesis and improved bile acid metabolism (190). Proteomic profiling revealed distinct hepatic expression patterns in the offspring of exercised dams, notably the upregulation of cytochrome P450 family 7 subfamily A member 1, a critical enzyme in cholesterol catabolism. Maternal physical activity has been proposed to stabilize the intrauterine environment through metabolic, endocrine and epigenetic pathways, thereby mitigating the susceptibility of offspring to MAFLD and other metabolic disorders (232). These findings suggest that exercise interventions during pregnancy offer a promising strategy for disrupting the intergenerational cycle of metabolic disease transmission. However, despite encouraging results from animal models, the precise epigenetic modifications involved, their persistence across generations, and their relevance in humans remain poorly understood. Understanding whether and how exercise-mediated epigenetic remodeling in the liver and germline can confer long-term protection against MAFLD across generations may provide valuable insights into refining preventive strategies. Although significant progress has been made in delineating the epigenetic mechanisms underlying the beneficial effects of exercise on MAFLD, substantial knowledge gaps remain. Future research integrating advanced epigenomic technologies, refined animal models and translational human studies is expected to play a pivotal role in uncovering the full therapeutic potential of exercise as an epigenetic intervention in metabolic liver diseases.

## 6. Conclusions

**Figure 2:**
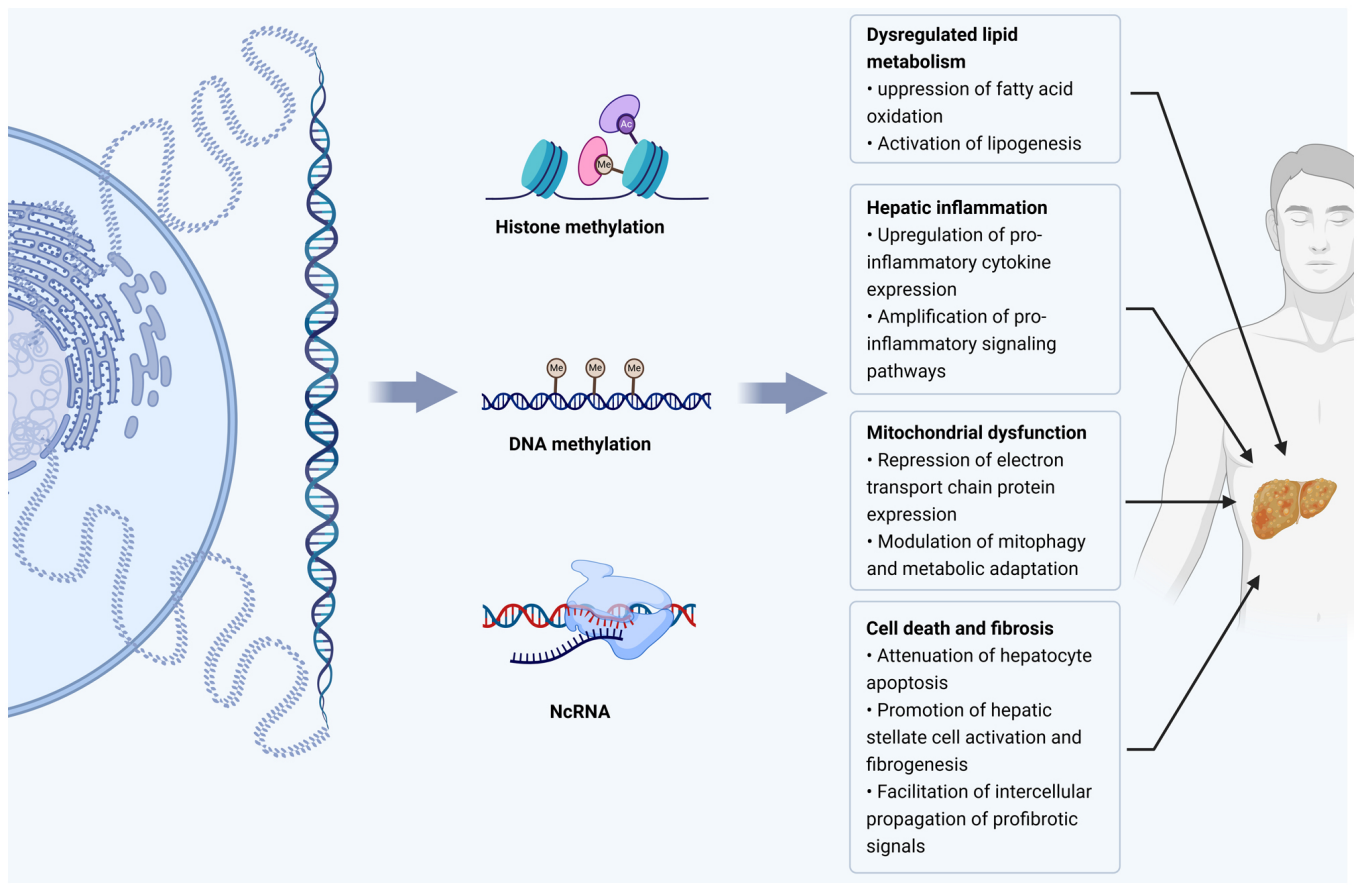
Physical activity exerts protective effects on metabolic dysfunction-associated fatty liver disease by modulating epigenetic regulatory pathways. NcRNA, non-coding RNA.

This review highlights the emerging role of exercise-induced epigenetic remodeling in MAFLD. Epigenetic dysregulation, which is characterized by altered DNA methylation landscapes, histone acetylation and methylation imbalances, and aberrant miRNA and lncRNA profiles, has been established as a central driver of hepatic lipid metabolic derangement, mitochondrial impairment, autophagic dysfunction, inflammatory activation, and fibrotic progression in MAFLD (Fig. 2). Exercise, as an effective non-pharmacological intervention, can reprogram these dysregulated epigenetic landscapes, thereby restoring mitochondrial function, reducing the hepatic lipid load, and alleviating systemic inflammation. Mechanistically, exercise modulates DNA methylation dynamics, reshapes histone modification landscapes, and reprograms the expression of ncRNAs, collectively promoting enhanced metabolic resilience. Despite these promising advances, substantial gaps remain in our understanding of exercise-induced epigenetic remodeling in MAFLD. Most available evidence is derived from animal models, which limits its translational applicability to human diseases. Further investigation with more robust clinical studies is urgently required to resolve this issue. Unraveling the epigenetic mechanisms underlying exercise-mediated hepatic protection could significantly advance the prevention and treatment of MAFLD.

## Figures and Tables

**Table I:** Exercise-induced epigenetic modifications and their functional implications in NAFLD.

First author/s, year	Epigenetic alterations	Sample type	Type of exercise	Effects of exercise	Gene	Action in NAFLD	(Refs.)
Pirola *et al*, 2013	DNA methylation	Human liver biopsy	Regular physical activity	Exercise reduces 5mC levels in mitochondrial DNA.	MT-ND6	Exercise improves mitochondrial function and alleviates liver fibrosis, inflammation, and tissue damage associated with MASH.	(92)
Zhou *et al*, 2017		Mouse liver tissue	Voluntary running	Exercise attenuated high-fat diet-induced hypermethylation.	SAA1/2, Il1r1, Hpn	Exercise mitigates hepatic lipid accumulation, inflammatory responses, and the activation of pro-tumorigenic transcriptional programs	(186)
Fan *et al*, 2024	Histone methylation	Mouse liver tissue	Moderate-intensity continuous training, High-intensity interval training	Exercise downregulates KMT2D, reducing H3K4me1 enrichment on IDI1 promoter.	IDI1	Exercise reduced liver steatosis, inflammation, cholesterol accumulation, and fibrosis.	(194)
Luo *et al*, 2024		Mouse liver tissue	Treadmill running	Exercise induces Klf10, suppressing H3K4me3 on Fh1promoters.	Fh1	Exercise alleviated MASH, reduced lipid accumulation, insulin resistance, inflammation, and fibrosis.	(195)
Lu *et al*, 2014	NcRNA	Mouse liver tissue	Hypoxic exercise training	Hypoxic training reduces microRNA-378, enhancing fatty acid oxidation.	CPT1A	Exercise improved lipid oxidation and metabolic profiles.	(219)
Xiao *et al*, 220	NcRNA	Mouse liver tissue	Treadmill running	Exercise suppresses microRNA-212, relieving inhibition of FGF21 to improve lipid metabolism.	FGF21	Exercise reduced liver lipid accumulation and injury.	(220)
de Mendonça *et al*, 2020		Human serum	Aerobic exercise	Exercise modulates circulating EV-miRNAs.	-	Exercise altered serum EV-miRNA profile, potential biomarkers.	(222)
Zhao *et al*, 2021		Mouse liver tissue	Aerobic exercise	Exercise upregulates microRNA-21a-5p to inhibit lipid accumulation genes.	FABP7, HMGCR, ACAT1, OLR1	Exercise improved lipid metabolism.	(227)
Wang *et al*, 2022		Mouse liver tissue	Aerobic exercise	Exercise suppresses microRNA-34a via ADAR2, alleviating hepatic lipid accumulation.	Srebp1c, Fasn	Exercise decreased hepatic TG and lipogenesis.	(223)
Wu *et al*, 2022		Mouse liver tissue	Aerobic exercise	Exercise inhibits lncRNA SRA, relieves suppression of ATGL via FoxO1.	FoxO1	Exercise reduced hepatic steatosis, inflammation.	(225)

5mC, 5-methylcytosine; ACAT1, acetyl-CoA acetyltransferase 1; ADAR2, adenosine deaminase acting on RNA 2; ATGL, adipose triglyceride lipase; CPT1A, carnitine palmitoyltransferase 1A; EV, extracellular vesicle; FABP7, fatty acid-binding protein 7; FASN, fatty acid synthase; FGF21, fibroblast growth factor 21; FH1, fumarate hydratase 1; FoxO1, forkhead box protein O1; H3K4me1, monomethylation of lysine 4 on histone H3; H3K4me3, trimethylation of lysine 4 on histone H3; HMGCR, 3-hydroxy-3-methylglutaryl-CoA reductase; IDI1, isopentenyl-diphosphate delta isomerase 1; Il1r1, interleukin 1 receptor type 1; Klf10, Krüppel-like factor 10; KMT2D, lysine methyltransferase 2D; lncRNA, long non-coding RNA; lncRNA SRA, steroid receptor RNA activator; MASH, metabolic dysfunction-associated steatohepatitis; MT-ND6, mitochondrially encoded NADH dehydrogenase 6; ncRNA, non-coding RNA; OLR1, oxidized low-density lipoprotein receptor 1; SAA1/2, serum amyloid A1/2; Srebp1c, sterol regulatory element-binding protein 1c.

## Data Availability

Not applicable.

## Abbreviations

5mC: 5-methylcytosine
AMPKα: AMP-activated protein kinase alpha
ChREBP: carbohydrate-responsive element-binding protein
circRNAs: circular RNAs
CpG: cytosine-phosphate-guanine
DNMTs: DNA methyltransferases
ER: endoplasmic reticulum
FGF-21: fibroblast growth factor-21
HATs: histone acetyltransferases
HDACs: histone deacetylases
HDMs: histone demethylases
HIIT: high-intensity interval training
HMTs: histone methyltransferases
HSC: hepatic stellate cell
KMT: lysine methyltransferase
lncRNAs: long non-coding RNAs
MAFLD: metabolic dysfunction-associated fatty liver disease
MALAT1: metastasis-associated lung adenocarcinoma transcript 1
MASH: metabolic dysfunction-associated steatohepatitis
miRNAs or miRs: microRNAs
MICT: moderate-intensity continuous training
mtDNA: mitochondrial DNA
MT-ND6: mitochondrially encoded NADH dehydrogenase 6
ncRNA: non-coding RNA
NF-κB: nuclear factor-kappa B
PGC-1α: peroxisome proliferator-activated receptor gamma coactivator 1-alpha
PPARα: peroxisome proliferator-activated receptor alpha
PTEN: phosphatase and tensin homolog
SIRTs: sirtuins
SREBP-1c: sterol regulatory element-binding protein-1c
TET: ten-eleven translocation
TNF-α: tumor necrosis factor-alpha
β-HAD: β-hydroxy-acyl-CoA dehydrogenase
